# Differential Localization of CEACAM-1 in Distinct Platelet Populations

**DOI:** 10.3390/cells15211920

**Published:** 2026-10-22

**Authors:** Natalia Bielicka, Adam Jan Olichwier, Tomasz Misztal, Magdalena Bruzgo-Grzybko, Izabela Suwda Kalita, Ewa Chabielska, Anna Gromotowicz-Popławska

**Affiliations:** 1 Department of Biopharmacy and Radiopharmacy, Medical University of Bialystok, 15-222 Bialystok, Poland; magdalena.bruzgo-grzybko@umb.edu.pl (M.B.-G.); ewa.chabielska@umb.edu.pl (E.C.); anna.gromotowicz@umb.edu.pl (A.G.-P.); 2 Radiopharmacy Centre, Medical University of Bialystok, 15-569 Bialystok, Poland; adam.olichwier@umb.edu.pl (A.J.O.); izabela.kalita@umb.edu.pl (I.S.K.); 3 Department of Physical Chemistry, Medical University of Bialystok, 15-089 Bialystok, Poland; tomasz.misztal@umb.edu.pl

**Keywords:** CEACAM-1, platelets, thrombosis, hemostasis, fibrin, clot, platelet populations

## Abstract

Carcinoembryonic antigen-related cell adhesion molecule-1 (CEACAM-1) is a platelet receptor recognized as a negative regulator of platelet activity. During thrombus formation, platelets differentiate into distinct populations, but whether CEACAM-1 is preferentially associated with specific platelet phenotypes remains unclear. Here, we investigated CEACAM-1 localization in different platelet populations, focusing on the accessibility of its extracellular domain. In platelets adherent to poly-L-lysine, the CEACAM-1 signal was predominantly intracellular and decreased following treatment with acetylsalicylic acid (ASA), whereas glucose increased this signal. During integrin-dependent spreading on fibrinogen, CEACAM-1 was undetectable by immunofluorescence, irrespective of αIIbβ3 inhibition with tirofiban, although its presence was confirmed by Western blotting. Flow cytometry revealed that ASA reduced the percentage of CEACAM-1-positive platelets following ADP stimulation under both normoglycemic and hyperglycemic conditions. Confocal microscopy demonstrated co-localization of CEACAM-1 with phosphatidylserine (PS)-positive regions, a hallmark of the procoagulant platelet phenotype. In thrombi formed on collagen fibers under flow, ASA reduced the CEACAM-1-positive area under both conditions. However, a reduction in the ratio of CEACAM-1-positive area to PS-exposing area was observed only under normoglycemic conditions. In fibrin–platelet clots, CEACAM-1 was detected only after prolonged clot incubation and subsequent platelet permeabilization. Collectively, these findings reveal differences in CEACAM-1 localization and detectability across platelet populations and experimental settings, suggesting an association with platelet functional specialization.

## 1. Introduction

Carcinoembryonic antigen-related cell adhesion molecule-1 (CEACAM-1) is a transmembrane glycoprotein expressed on platelets, endothelial cells, and various immune cells. As a member of the immunoglobulin superfamily, CEACAM-1 participates in intercellular adhesion and cell–cell interactions. Its extracellular domain mediates homophilic receptor interactions. Because these interactions depend on domain accessibility, changes in its spatial organization may influence CEACAM-1 function. Alternative splicing involving exon 7 generates isoforms with either a long (CEACAM-1L; 71–73 amino acids) or a short (CEACAM-1S; 10 amino acids) cytoplasmic tail. CEACAM-1L contains an immunoreceptor tyrosine-based inhibitory motif (ITIM)-containing signalling domain, whereas CEACAM-1S lacks the ITIM motif [1]. In platelets, phosphorylation of the ITIM motif recruits SH2 domain-containing phosphatases that counteract signalling mediated by immunoreceptor tyrosine-based activation motif (ITAM)-bearing receptors, particularly the collagen receptor complex GPVI/FcRγ, thereby limiting platelet activation. Thus, ITIM-dependent signalling attributed to CEACAM-1 is specifically associated with the CEACAM-1L isoform [2,3].

To date, the functions of CEACAM-1 in platelets have been investigated primarily using knockout models. In these studies, platelets from *Ceacam1^−^/^−^* mice exhibit enhanced activity and thrombus formation [4]. Despite its inhibitory role, CEACAM-1 also promotes αIIbβ3-dependent responses, including cytoskeletal reorganization, spreading on fibrinogen, and clot retraction [5]. However, the distribution of CEACAM-1 among distinct platelet populations has remained largely unexplored. This is relevant because platelets undergo functional specialization during activation. As the localization of platelet markers often reflects their function [6], we investigated CEACAM-1 across distinct platelet populations exposed to different extracellular matrices and microenvironments with particular attention to the accessibility of its extracellular domain. Experimental models representing these platelet activation states included platelets in suspension, adhesion to poly-L-lysine (PLL), spreading on fibrinogen, thrombus formation on collagen under flow, and platelet incorporation into fibrin clots. Platelet function was pharmacologically modulated using acetylsalicylic acid (ASA), a reference antiplatelet agent with well-established inhibitory effects on platelet activation and thrombus formation [7], whereas tirofiban [8], an αIIbβ3 inhibitor, was applied in αIIbβ3-dependent models of platelet spreading on fibrinogen and platelet–fibrin interactions during clot formation. Acute exposure to elevated glucose was used to assess CEACAM-1 under conditions of enhanced platelet activation induced by high glucose [9].

## 2. Materials and Methods

The human and murine experiments were designed as complementary, model-specific analyses rather than as a direct cross-species comparison. Mouse blood was used to assess platelet responses following in vivo ASA exposure in flow cytometric and collagen-flow assays. Human platelets from healthy donors were used for ex vivo localization studies, fibrin-platelet clot formation, spreading on fibrinogen, and Western blot analysis. 

### 2.1. Blood Collection for Human Study

The study protocol was reviewed and accepted by the Bioethics Committee of the Medical University of Bialystok (Approval No.: APK.002.103.2023, date of approval: 16 February 2023). After obtaining informed consent, in accordance with the Declaration of Helsinki, blood samples were collected from healthy volunteers in vacutainers containing sodium citrate as an anticoagulant. 

Platelet-rich plasma (PRP), platelet-poor plasma (PPP), and washed platelets were prepared as previously described [10].

### 2.2. Animals

Experiments were conducted in accordance with the EU Guidelines on Animal Experiments (European Directive 2010/63/EU), and all procedures involving animals and their care were approved by the Local Ethical Committee on Animal Testing in Olsztyn (Approval No.: 8/2023, date of approval: 18 January 2023). The animals were purchased from the Centre for Experimental Medicine in Bialystok.

Experiments were conducted in male wild-type C57BL/6J mice weighing 23–25 g. Before blood collection, animals were anaesthetized intraperitoneally with ketamine (120 mg/kg, Ketamina 10%; Biowet, Puławy, Poland) and xylazine (12.5 mg/kg, 12.5 mg/kg; Xylapan, Biowet, Puławy, Poland). Blood was withdrawn from the right cardiac ventricle into 3.8% sodium citrate (1:10, *v*/*v*), after which the animals were euthanized by cervical dislocation. 

### 2.3. Acetylsalicylic Acid Administration

Fifteen minutes before blood collection, ASA (Flectadol, 1 g/5 mL; Sanofi, Paris, France) was administered through the left femoral vein at 3, 10, or 30 mg/kg in a final injection volume of 0.1 mL. ASA was prepared in PBS, whereas control mice received the corresponding volume of PBS alone. The ASA doses were selected based on the pharmacologically active range previously established in a mouse model [7]. Control animals received an equal volume of PBS. Collected blood samples were subsequently used for flow cytometry analysis and for thrombus formation experiments performed under flow conditions.

### 2.4. Flow Cytometric Analysis of Platelet Populations

CytoFLEX flow cytometer (Model A00-1-1102, Beckman Coulter Inc., Brea, CA, USA) equipped with CytExpert 2.4 software was utilized. Blood samples were incubated with glucose (final concentration: 20 mM) or vehicle (0.9% NaCl) for 20 min. Mouse blood was centrifuged at 129× *g* for 10 min at room temperature to obtain PRP. The platelet count in the supernatant was adjusted to 10 × 10^7^ cells/mL with autologous PPP using a ScilVet ABC Plus+ haematological analyzer (HORIBA ABX, Palaiseau, France). The adjusted PRP was supplemented with the thrombin inhibitor D-phenylalanyl-l-prolyl-l-arginine chloromethyl ketone (PPACK; final concentration: 40 µM; Santa Cruz Biotechnology, Dallas, TX, USA) and calcium chloride (CaCl_2_; final concentration: 2 mM). Platelets were then activated with adenosine diphosphate (ADP, final concentration: 20 µM; Chrono-Log Corp., Havertown, PA, USA) and incubated for 10 min. During the final 5 min of agonist incubation, PRP was incubated with DyLight 488-labelled anti-CD42b antibody (glycoprotein Ib, GP Ib; final concentration: 4.44 µg/mL; Emfret Analytics, Eibelstadt, Germany) for platelet identification. In separate samples, Alexa Fluor 647-labelled annexin V (ANX V; final concentration: 5 µg/mL; Thermo Fisher Scientific, Waltham, MA, USA) was used to detect phosphatidylserine (PS) exposure, whereas anti-CEACAM-1 antibody (final concentration: 3 µg/mL; R&D Systems, Minneapolis, MN, USA) was used to assess CEACAM-1 surface availability. The immunogen used to generate this antibody was recombinant mouse CEACAM-1 encompassing residues Met1–Gly428 (UniProtKB accession P31809). Accordingly, the percentages of ANX V-positive and CEACAM-1-positive platelets were determined independently in separate samples. In unstimulated samples, two platelet populations differing in GP Ib fluorescence were distinguishable (Figure S1). Following ADP stimulation, a distinct platelet population characterized by increased APC fluorescence became apparent. In ANX V-stained samples, platelets within this population were identified as ANX V-positive, PS-exposing procoagulant platelets. In parallel, in anti-CEACAM-1-stained samples, the platelet population exhibiting high APC fluorescence was classified as CEACAM-1-positive. The percentages of ANX V-positive and CEACAM-1-positive platelets within the total platelet population were used for statistical analysis. To assess background fluorescence in the APC channel, ADP-stimulated platelets stained with GP Ib, but without the anti-CEACAM-1 antibody or ANX V, were examined in separate control samples (Figure S2). In these controls, the population with the highest GP Ib fluorescence retained low APC fluorescence, broadly overlapping with that of the two populations with lower GP Ib fluorescence. These observations support the identification of the APC-high populations in the corresponding stained samples as CEACAM-1-positive or ANX V-positive, respectively.

### 2.5. Confocal Microscopy Imaging

Confocal microscopy was carried out on a Zeiss Axio Examiner Z1 fixed-stage microscope (Carl Zeiss Microscopy GmbH, Oberkochen, Germany) coupled to a CSU-X1 spinning-disk confocal scanner (Yokogawa Electric Corporation, Tokyo, Japan). A W Plan-Apochromat 20×/1.0 water-immersion objective (Carl Zeiss Microscopy GmbH, Oberkochen, Germany) was used for image acquisition. Images were acquired and analysed with SlideBook 6.0 software (Intelligent Imaging Innovations, Inc., Denver, CO, USA).

For imaging of activated human platelets, washed platelets were incubated with ADP (20 µM) and CaCl_2_ (2 mM) for a total of 15 min. Alexa Fluor 647-conjugated anti-CEACAM-1 antibody (3 µg/mL) and Alexa Fluor 488-labelled ANX V (5 µg/mL) were added during the final 5 min of incubation. The platelet suspensions were then transferred to µ-Slide VI 0.4 chambers (Ibidi, Gräfelfing, Germany) and examined by confocal microscopy.

### 2.6. Assessment of CEACAM-1 and ANX V in Thrombi Formed on Collagen Under Flow Conditions

Thrombi were generated on coverslips coated with type I collagen (50 µg/mL; Horm collagen, Nycomed, Zurich, Switzerland). Prior to perfusion, blood samples were incubated for 30 min with either glucose (20 mM) or vehicle (0.9% NaCl). To inhibit coagulation, whole blood was supplemented with PPACK (40 µM; Santa Cruz Biotechnology, Dallas, TX, USA), CaCl_2_ (3 mM), and MgCl_2_ (3 mM). Blood was then perfused over the collagen-coated surface for 4 min at a wall shear rate of 1000 s^−1^, representing arterial flow conditions. After perfusion, the thrombus-containing surface was rinsed with HEPES buffer and removed from the chamber. Thrombi were subsequently stained for 5 min with anti-CEACAM-1 antibody (3 µg/mL) together with Alexa Fluor 488-conjugated ANX V (5 µg/mL; Thermo Fisher Scientific, Waltham, MA, USA). Excess staining reagents were removed by washing with HEPES buffer, after which end-stage thrombi were examined by confocal microscopy. Images were analysed using SlideBook 6.0 software. The CEACAM-1-positive area was divided by the ANX V-positive area, and the resulting value was expressed as the CEACAM-1^+^ area/ANX V^+^ area ratio.

### 2.7. Assessment of CEACAM-1 Localization and PS Exposure in Fibrin-Platelet Clots

Human PRP samples were incubated with glucose (final concentration: 20 mM) or vehicle (0.9% NaCl) for 15 min, followed by incubation with tirofiban (0.05, 0.5, or 5 µg/mL) for an additional 15 min. Subsequently, Alexa Fluor 488-conjugated human fibrinogen (Thermo Fisher Scientific, Waltham, MA, USA) was added to PRP (final concentration: 15 µM), and clot formation was induced by the addition of CaCl_2_ (final concentration: 15 mM). Samples were transferred into Ibidi μ-Slides VI 0.4 and incubated at 37 °C for 2 h. 

Fibrin net density was quantified from confocal images of the formed clots. For each image, four 40-µm lines were positioned at random locations, and the number of fibrin fibers crossing each line was recorded. The average number of intersections was used as a measure of relative fibrin net density. To assess PS exposure, Alexa Fluor 647-labeled ANX V (final concentration: 5 µg/mL) diluted in PBS was added to the formed clots and incubated for 5 min prior to confocal imaging. The same procedure was performed using Alexa Fluor 647-conjugated anti-CEACAM-1 antibody (FAB2244R; final concentration: 3 µg/mL; R&D Systems, Minneapolis, MN, USA) to assess extracellular CEACAM-1 localization. The immunogen used to generate this antibody was recombinant human CEACAM-1 encompassing residues Gln35–Gly428 (UniProtKB accession P13688).

To evaluate intracellular CEACAM-1 localization, clots were incubated overnight at room temperature, fixed with 1% paraformaldehyde (PFA; Warchem, Zakręt, Poland), and washed with PBS. Samples were subsequently permeabilized and stained by incubation with a mixture of 0.2% saponin (Sigma-Aldrich, Darmstadt, Germany), 0.1% bovine serum albumin (BSA, Sigma-Aldrich, Darmstadt, Germany) and anti-CEACAM-1 antibody for 1 h at room temperature. Unbound antibody was removed by washing with PBS, and samples were analysed by confocal microscopy.

### 2.8. Platelet Adhesion on Poly-L-Lysine (PLL) and CEACAM-1/GP Ib Ratio Assessment

A previously described protocol was used with minor modifications [10]. Washed human platelets were adjusted to a concentration of 250 × 10^6^/mL and incubated with glucose (20 mM) for 15 min followed by a 15-min incubation with ASA at final concentrations of 0.18, 1.8, or 9 µg/mL [7] for 15 min. In parallel, an additional set of samples was treated with the same concentrations of ASA in the absence of glucose. Samples were stained with an Alexa Fluor 488-conjugated anti-GP Ibα antibody (final concentration: 1.3 µg/mL; BioLegend, San Diego, CA, USA), used as a platelet marker, and fixed with 1% PFA for 15 min. Fixed samples were centrifuged (2200× *g*, 2 min), resuspended in PBS containing BSA, centrifuged again (2200× *g*, 2 min), and permeabilized with PBS containing 0.1% saponin for 10 min. Subsequently, platelets were incubated with anti-CEACAM-1 antibody (final concentration: 3 µg/mL) for 30 min. Samples were then centrifuged (2200× *g*, 2 min), resuspended in PBS containing 0.1% saponin, centrifuged again (2200× *g*, 2 min), and finally resuspended in PBS containing 0.1% BSA before immediate adhesion to PLL-coated surfaces. Samples were imaged by confocal microscopy. Image analysis was performed using SlideBook 6.0 software. The CEACAM-1-positive area was divided by the GP Ib-positive area, and the resulting value was expressed as the CEACAM-1^+^ area/GP Ib^+^ area ratio. 

### 2.9. Platelet Spreading on Fibrinogen

The procedure was performed as previously described with modifications [11]. Briefly, washed human platelets were adjusted to a concentration of 100 × 10^6^/mL and incubated with tirofiban (final concentrations: 0.05, 0.5, or 5 µg/mL) for 20 min. Subsequently, CaCl_2_ was added (final concentration: 2 mM), and platelets were allowed to adhere to glass coverslips precoated with fibrinogen (100 µg/mL; Sigma-Aldrich, Darmstadt, Germany). After 5 min of initial adhesion, platelets were either left untreated or stimulated with ADP (final concentration: 20 µM) or thrombin receptor-activating peptide-6 (TRAP-6; final concentration: 15 µM) and incubated for an additional 40 min.

Samples were fixed with 1% PFA for 15 min and washed three times with PBS. Subsequently, platelets were permeabilized with 0.005% sodium dodecyl sulfate (SDS; Warchem, Zakręt, Poland) in PBS and washed three times with PBS. Non-specific binding was blocked with 5% BSA. The samples were subsequently labelled for 1 h at room temperature with Alexa Fluor 488-conjugated anti-GP Ibα antibody (final concentration: 1.5 µg/mL) and Alexa Fluor 647-conjugated anti-CEACAM-1 antibody (final concentration: 3 µg/mL).

Samples were imaged by confocal microscopy. Image analysis was performed using SlideBook 6.0 software. GP Ib fluorescence was used to define the area covered by spread platelets.

### 2.10. Protein Quantification and Western Blot Analysis

Following 45 min of platelet spreading on fibrinogen, samples were incubated with 1% Triton X-100 for 10 min to solubilize platelet membranes and release membrane-associated CEACAM-1. The resulting extract was collected and operationally designated the Triton X-100-soluble membrane-associated fraction. The remaining Triton X-100-insoluble material was washed with PBS and subsequently incubated with 2% SDS preheated to 70 °C for 15 min. The resulting extract was designated the Triton X-100-insoluble/SDS-solubilized, cytoskeletal-enriched fraction. For Western blot analysis, the Triton X-100-soluble and Triton X-100-insoluble/SDS-solubilized fractions were combined to obtain a single protein extract from spread platelets, which was used to assess the presence of CEACAM-1. Unstimulated washed platelets were additionally included in the analysis as a reference sample. Protein concentrations were determined using the Pierce™ 660 nm Protein Assay (22660, Thermo Fisher Scientific, Waltham, MA, USA) with Pre-Diluted Protein Assay Standards, Bovine Serum Albumin (BSA) Set (23208, Thermo Fisher Scientific, Waltham, MA, USA), according to the manufacturer’s instructions. Protein samples were concentrated by precipitation with four volumes of ice-cold acetone for 2 h at −20 °C, followed by centrifugation at 13,000–15,000× *g* for 10 min. Pellets were briefly air-dried, resuspended in 2× Laemmli sample buffer (#161-0737, Bio-Rad, Hercules, CA, USA), and denatured at 95 °C for 5 min. Proteins were separated on Mini-PROTEAN TGX Stain-Free 4–15% gels (#4568084, Bio-Rad, Hercules, CA, USA), transferred onto PVDF membranes (Trans-Blot Turbo Mini-size LF PVDF Membrane, #10026934, Bio-Rad, Hercules, CA, USA), and blocked with EveryBlot Blocking Buffer (#12010020, Bio-Rad, Hercules, CA, USA). Membranes were incubated with appropriate primary, Anti-human CEACAM-1 antibody (R&D Systems, Minneapolis, MN, USA; Cat. No. FAB2244R) and HRP-conjugated rat anti-mouse IgG secondary antibody (Bio-Rad, Hercules, CA, USA; LO-ME-3) and immunoreactive bands were detected using Clarity Max ECL substrate (#1705062, Bio-Rad, Hercules, CA, USA) and visualized with a ChemiDoc Imaging System (Bio-Rad, Hercules, CA, USA). Ponceau S staining (Thermo Scientific, Waltham, MA, USA, A40000279) was used to assess protein loading.

### 2.11. Statistical Analysis

Statistical analyses were performed using GraphPad Prism 9.4.1 (GraphPad Software, San Diego, CA, USA). Data distribution was assessed using the Shapiro–Wilk test. Normally distributed data were analysed using one-way ANOVA followed by an appropriate post hoc test. For non-normally distributed data, the Kruskal–Wallis test followed by Dunn’s multiple comparisons test was used. Data are presented as mean ± standard error of the mean (SEM), with individual data points representing independent determinations (*n*). A *p*-value < 0.05 was considered statistically significant.

### 2.12. Use of Artificial Intelligence Tools

Generative artificial intelligence tools were used to assist in language editing, text revision, and improvement of readability. No artificial intelligence tools were used for data generation, analysis, interpretation, or scientific decision-making.

## 3. Results

### 3.1. Flow Cytometric Analysis of Platelet Populations

**Figure 1:**
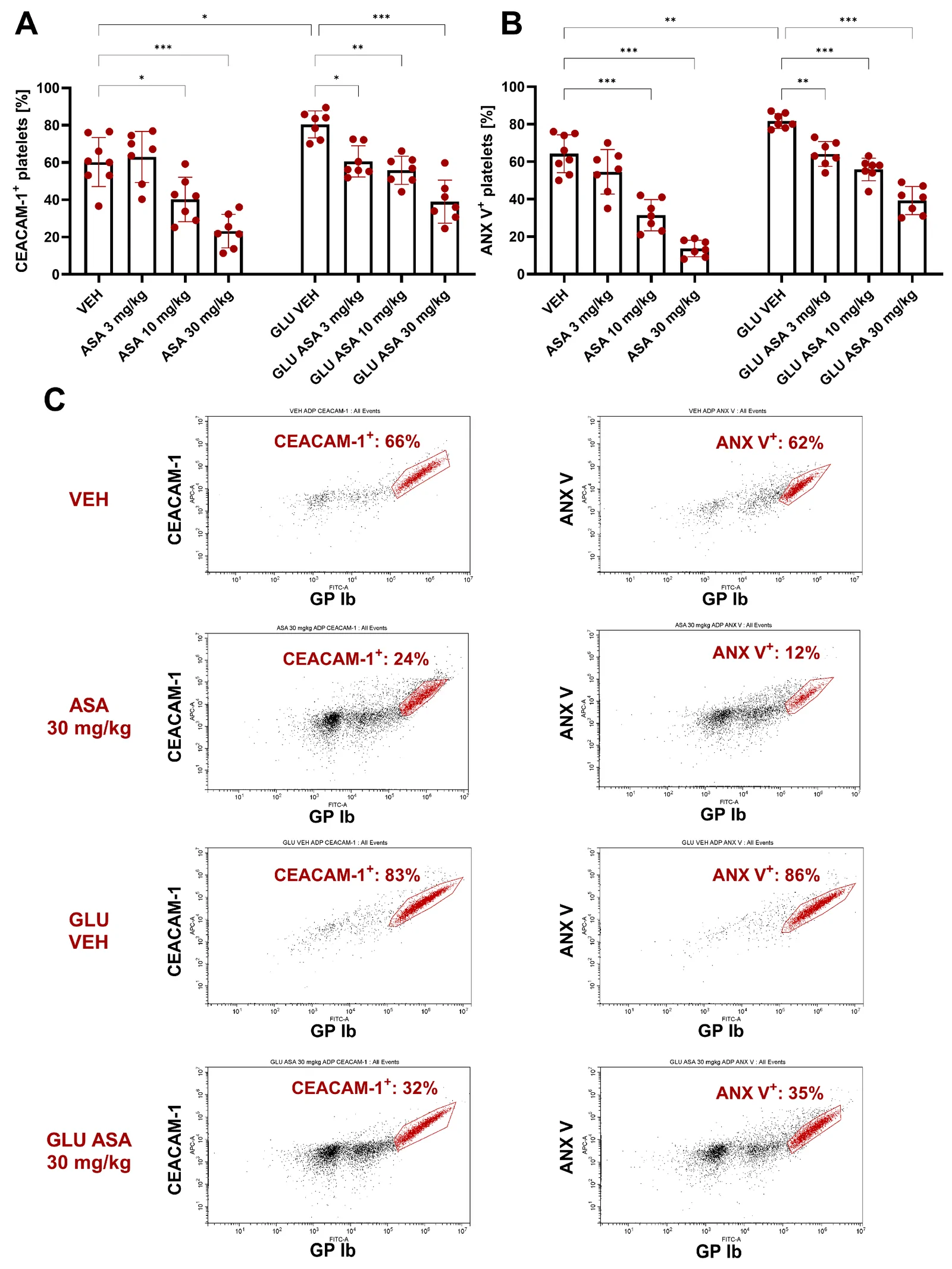
**Flow cytometric quantification of CEACAM-1-positive (CEACAM-1^+^) and ANX V-positive (ANX V^+^) platelets in ADP-activated platelets.** ANX V^+^ reflects PS exposure. (**A**) Reduction in the percentage of CEACAM-1^+^ platelets after ASA treatment under normoglycemic and hyperglycemic (GLU) conditions. * *p* < 0.05, ** *p* < 0.01, *** *p* < 0.001; *n* = 7–8 mice per group. One measurement per mouse was included in the statistical analysis, with each mouse representing one biological replicate. Statistical significance was determined by one-way ANOVA with Tukey’s post hoc multiple comparisons test. (**B**) Reduction in the percentage of ANX V^+^ platelets after ASA treatment under normoglycemic and hyperglycemic (GLU) conditions. ** *p* < 0.01, *** *p* < 0.001; *n* = 7–8 mice per group. One measurement per mouse was included in the statistical analysis, with each mouse representing one biological replicate. Statistical significance was determined by one-way ANOVA with Tukey’s post hoc multiple comparisons test. (**C**) Representative dot plots showing ADP-activated platelets. The horizontal axis shows GP Ib fluorescence in the FITC channel, whereas the vertical axis shows CEACAM-1 (dot plots on the left) or ANX V (dot plots on the right) fluorescence in the APC channel. Populations with increased APC fluorescence were classified as CEACAM-1^+^ or ANX V^+^ platelets, respectively, and are highlighted in red. Percentages of CEACAM-1^+^ and ANX V^+^ populations are indicated on the respective dot plots. Experimental groups are indicated to the left of the dot plots. Each pair of dot plots (dot plot with CEACAM-1 fluorescence and dot plot with ANX V fluorescence) within an experimental group represents an independent experiment using platelets from a single mouse. The corresponding control samples are shown in Figure S1.

Procoagulant platelets are characterized by the externalization of PS on the platelet surface. ANX V binds to exposed PS and was therefore used to identify platelets exhibiting a procoagulant phenotype. Confocal microscopy imaging of procoagulant platelets revealed a spatial association between CEACAM-1-positive and ANX V-positive, PS-exposing regions (Figure S3). Based on this observation, we next examined by flow cytometry whether the percentages of CEACAM-1-positive and ANX V-positive platelets were similarly modulated under conditions associated with decreased or enhanced platelet activity. ASA was selected as a reference antiplatelet agent to reduce platelet activity, whereas high-glucose conditions were used to examine platelet responses under elevated extracellular glucose, which is associated with enhanced platelet activity. Platelets were analyzed in suspension, with limited stable platelet–platelet interactions. Under normoglycemic conditions, ASA at doses of 10 and 30 mg/kg reduced the percentage of CEACAM-1-positive platelets (Figure 1A). Similarly, ASA at doses of 10 and 30 mg/kg reduced the percentage of ANX V-positive platelets (Figure 1B). Glucose treatment increased the percentages of both CEACAM-1-positive (Figure 1A) and ANX V-positive (Figure 1B) platelets. Under hyperglycemic conditions, all three ASA doses (3, 10, and 30 mg/kg) reduced the percentages of both CEACAM-1-positive and ANX V-positive platelets (Figure 1A,B). Together, these findings indicate that high-glucose conditions increased, whereas ASA reduced, the percentage of CEACAM-1-positive platelets following ADP stimulation.

### 3.2. Assessment of CEACAM-1 and PS Exposure in Thrombi Formed on Collagen Under Flow Conditions

In contrast to the platelet suspension model (Section 3.1.), the flow chamber model involved platelet adhesion to collagen fibres and platelet–platelet interactions leading to thrombus formation. Because collagen is a potent platelet agonist, this model induces strong platelet activation, including PS exposure. Importantly, under these experimental conditions, thrombi are platelet-rich and form in the absence of fibrin generation. In this model, we assessed CEACAM-1-positive and ANX V-positive areas within thrombi. Because CEACAM-1 and ANX V were measured in the same thrombi, we calculated the CEACAM-1^+^ area/ANX V^+^ area ratio to assess how the two signals changed relative to each other. As in Section 3.1, the same ASA and high-glucose treatments were applied to assess CEACAM-1 and ANX V during thrombus formation under flow.

**Figure 2:**
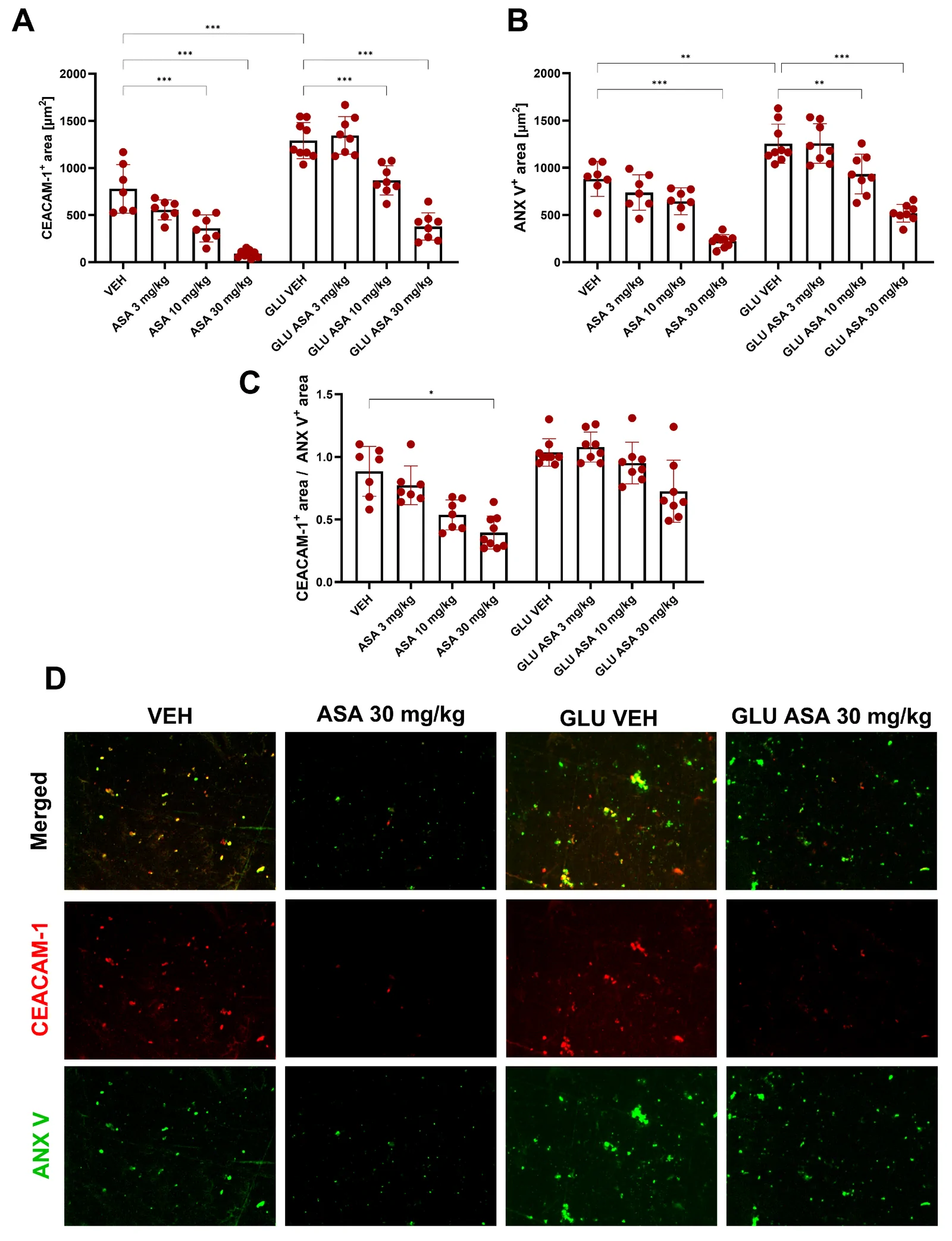
**Changes in CEACAM-1-positive (CEACAM-1^+^) and ANX V-positive (ANX V^+^) areas in thrombi formed on collagen fibers under flow.** ANX V^+^ reflects PS exposure. (**A**) Changes in the area of CEACAM-1^+^ platelets in thrombi after ASA treatment under normoglycemic and hyperglycemic (GLU) conditions. *** *p* < 0.001; *n* = 7–9 mice per group. For each mouse, thrombus formation on collagen was induced once, with each mouse representing one biological replicate. Statistical significance was determined by one-way ANOVA with Tukey’s post hoc multiple comparisons test. (**B**) Changes in the area of ANX V^+^ platelets in thrombi after ASA treatment under normoglycemic and hyperglycemic (GLU) conditions. ** *p* < 0.01, *** *p* < 0.001; *n* = 7–9 mice per group. For each mouse, thrombus formation on collagen was induced once, with each mouse representing one biological replicate. Statistical significance was determined by one-way ANOVA with Tukey’s post hoc multiple comparisons test. (**C**) Changes in CEACAM-1^+^ area/ANX V^+^ area ratio after ASA treatment under normoglycemic and hyperglycemic (GLU) conditions. * *p* < 0.05; *n* = 7–9 mice per group. For each mouse, thrombus formation on collagen was induced once, with each mouse representing one biological replicate. Statistical significance was determined by Kruskal–Wallis test with Dunn’s multiple comparisons test. (**D**) Representative confocal images showing CEACAM-1 (red) and PS exposure assessed by ANX V (green) within thrombi formed on collagen fibers under flow conditions. Each representative image corresponds to a single field of view obtained from one mouse. For statistical analysis, one field of view was analyzed per mouse, with each mouse considered one biological replicate. From left to right, the columns represent VEH, ASA 30 mg/kg, GLU VEH, and GLU ASA 30 mg/kg, respectively. From top to bottom, the rows show merged images, CEACAM-1 fluorescence (red), and ANX V fluorescence (green), respectively. Magnification ×400.

Under normoglycemic conditions, ASA at doses of 10 and 30 mg/kg reduced the CEACAM-1-positive area (Figure 2A), whereas ASA at 30 mg/kg reduced the ANX V-positive area (Figure 2B). Glucose increased both CEACAM-1-positive (Figure 2A) and ANX V-positive (Figure 2B) areas. Under hyperglycemic conditions, ASA at doses of 10 and 30 mg/kg reduced both CEACAM-1-positive (Figure 2A) and ANX V-positive (Figure 2B) areas. ASA reduced the CEACAM-1^+^ area/ANX V^+^ area ratio only at 30 mg/kg under normoglycemic conditions. Glucose treatment did not affect this ratio (Figure 2C). These findings suggest that ASA-induced reductions in CEACAM-1-positive and PS-exposing areas within thrombi are not necessarily proportional, as reflected by the decreased area ratio at 30 mg/kg under normoglycemic conditions. Glucose increased both areas, but neither glucose alone nor ASA treatment under hyperglycemic conditions detectably altered their ratio.

### 3.3. Assessment of CEACAM-1 Localization and PS Exposure in Fibrin-Platelet Clots

**Figure 3:**
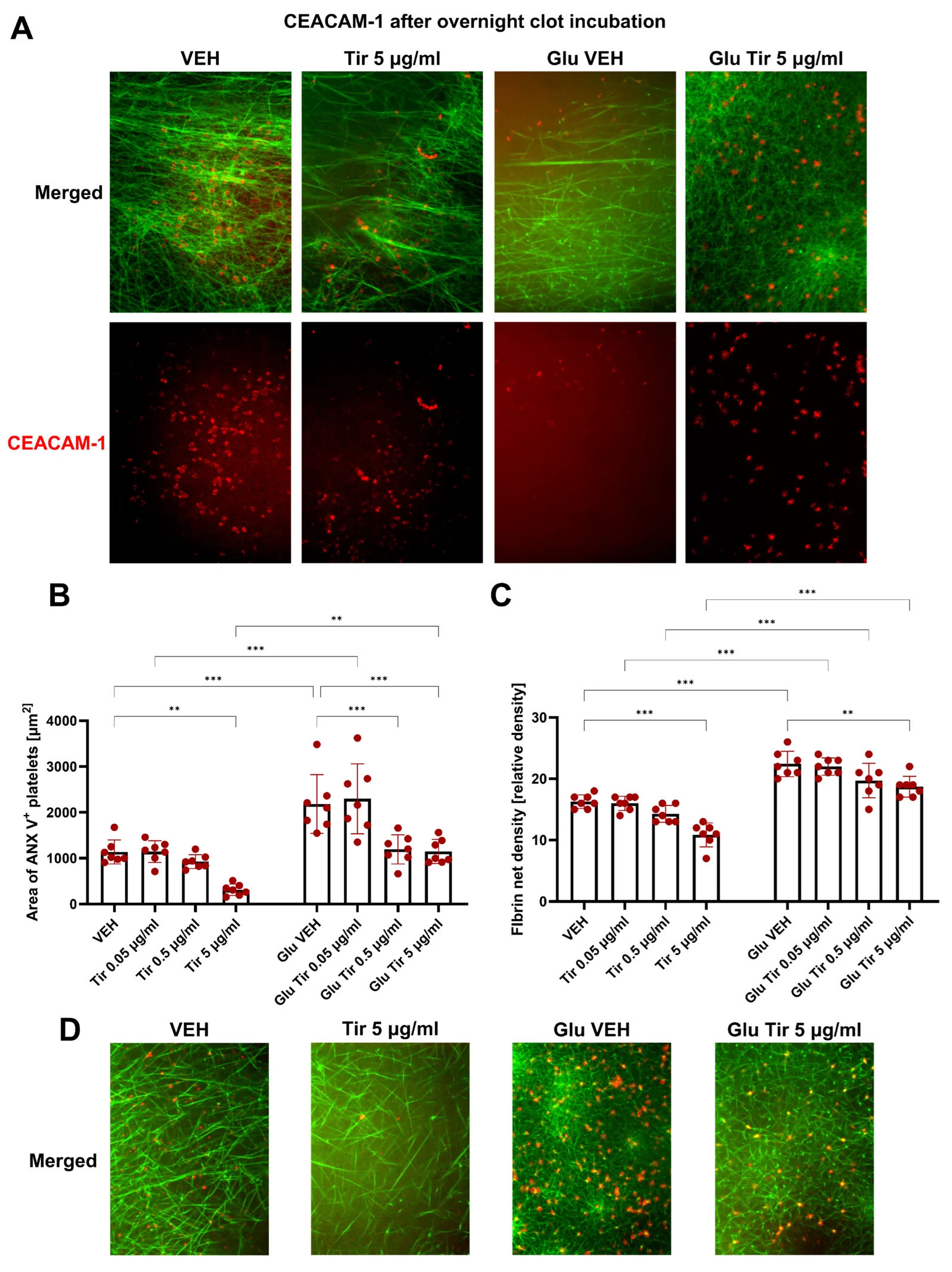
**CEACAM-1 localization and tirofiban (Tir)-dependent changes in PS exposure and fibrin net density in fibrin–platelet clots under normoglycemic and hyperglycemic (GLU) conditions.** ANX V^+^ reflects PS exposure. (**A**) Representative confocal images of fibrin–platelet clots after overnight incubation, permeabilization, and CEACAM-1 staining. The top row shows merged images of fibrin fibers (green) and CEACAM-1 fluorescence (red), whereas the bottom row shows CEACAM-1 fluorescence alone (red). Columns represent the experimental groups, as indicated above the images. In comparison with the fibrin net shown in panel (**D**), overnight-incubated clots exhibited heterogeneous morphology, including areas of partial fibrin lysis, clot contraction, and regions with relatively preserved fibrin structure. Similar patterns were observed in all experimental groups. Representative images obtained from different clot regions are presented to illustrate the structural heterogeneity of the clots. Magnification ×400. (**B**) Changes in PS exposure, expressed as ANX V^+^ area, after tirofiban (Tir) treatment under normoglycemic and hyperglycemic (GLU) conditions. ** *p* < 0.01, *** *p* < 0.001; *n* = 7 healthy volunteers per group. Plasma obtained from one volunteer was used to prepare 16 fibrin–platelet clots (1 clot per experimental group for ANX V or CEACAM-1 staining), and each clot represented one biological replicate. Statistical significance was determined by one-way ANOVA with Tukey’s post hoc multiple comparisons test. (**C**) Changes in fibrin net density after tirofiban (Tir) treatment under normoglycemic and hyperglycemic (GLU) conditions. ** *p* < 0.01, *** *p* < 0.001; *n* = 7 healthy volunteers per group. Plasma obtained from one volunteer was used to prepare 16 fibrin–platelet clots (1 clot per experimental group for ANX V or CEACAM-1 staining), and each clot represented one biological replicate. Statistical significance was determined by one-way ANOVA with Tukey’s post hoc multiple comparisons test. (**D**) Merged images containing both ANX V and fibrin fluorescence are shown. Representative confocal images illustrate PS exposure within fibrin–platelet clots, assessed by ANX V binding. ANX V fluorescence is shown in red, whereas fibrin fibers are shown in green. Each column represents a separate experimental group, as indicated above the images. Magnification ×400.

In contrast to the flow chamber model, the fibrin–platelet clot model involved platelets incorporated into a fibrin net. In this model, we assessed CEACAM-1 localization and ANX V-positive, PS-exposing procoagulant platelets within the clots. Tirofiban was used to inhibit integrin αIIbβ3, which mediates platelet–fibrin interactions central to this model whereas, as in the previous model, high-glucose conditions were used to enhance platelet activity. Fibrin net structure was also evaluated under these conditions. In freshly formed clots, CEACAM-1 fluorescence was not detected either before or after platelet permeabilization (Figure S4). However, following overnight clot incubation and subsequent permeabilization, CEACAM-1 staining became detectable within platelets embedded in fibrin-platelet clots (Figure 3A). Similar staining patterns were observed in all experimental groups. Owing to the heterogeneous structure of lysed and contracted clots and the uneven distribution of platelets within the fibrin net, quantitative analysis was not performed. Platelets exhibiting CEACAM-1 staining displayed an irregular morphology with pseudopodia-like extensions (Figure 3A), whereas ANX V-positive procoagulant platelets showed a rounded morphology (Figure 3D). Tirofiban treatment reduced PS exposure, expressed as a decreased area of ANX V-positive platelets (Figure 3B) and decreased fibrin net density (Figure 3C). These findings show that in fibrin–platelet clots, CEACAM-1 becomes detectable only after prolonged clot incubation and platelet permeabilization, whereas αIIbβ3 inhibition did not alter this pattern of CEACAM-1 detection.

### 3.4. Platelet Adhesion on Poly-L-Lysine and CEACAM-1/GP Ib Ratio Assessment

**Figure 4:**
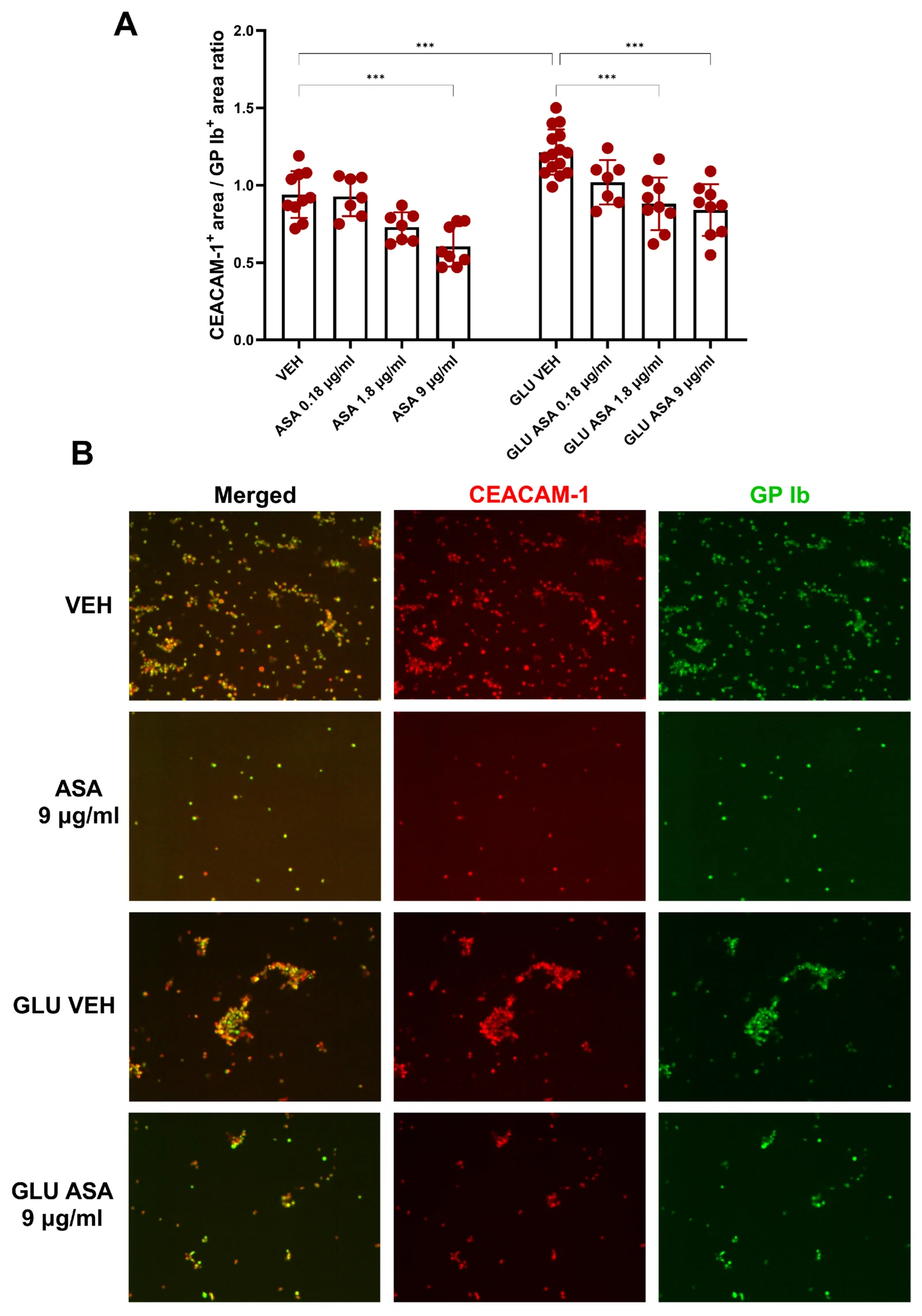
**CEACAM-1^+^ area/GP Ib^+^ area ratio in platelets adhered to poly-L-lysine (PLL).** (**A**) Changes in the CEACAM-1^+^ area/GP Ib^+^ area ratio after ASA treatment under normoglycemic and hyperglycemic (GLU) conditions. *** *p* < 0.001; *n* = 7–10 healthy volunteers per group, washed platelets from one healthy volunteer were used to prepare 8 samples (1 sample per 1 experimental group); one field of view per sample was analyzed, and each volunteer represented one biological replicate. Statistical significance was determined by one-way ANOVA with Tukey’s post hoc multiple comparisons test. (**B**) Representative confocal images of platelets adhered to PLL-coated surfaces. From left to right, the columns show merged images containing CEACAM-1 (red) and GPIb (green) fluorescence, CEACAM-1 fluorescence alone (red), and GP Ib fluorescence alone (green), respectively. Each row represents a separate experimental group indicated to the left of the images.

In the PLL adhesion model, platelets did not expose PS and retained a predominantly rounded morphology, consistent with a low-activation, non-procoagulant phenotype. We therefore used this model to examine CEACAM-1 localization in platelets in the absence of the PS-exposing procoagulant phenotype observed in the previous experimental models. CEACAM-1 fluorescence was assessed relative to GP Ib, which was used as a platelet marker, and expressed as the CEACAM-1^+^ area/GP Ib^+^ area ratio. ASA and high-glucose treatments were applied to examine CEACAM-1 under conditions associated with reduced and enhanced platelet activity, respectively. Under normoglycemic conditions, ASA reduced the CEACAM-1^+^ area/GP Ib^+^ area ratio at 9 µg/mL, whereas under hyperglycemic conditions a reduction was observed at 1.8 and 9 µg/mL Glucose increased the CEACAM-1^+^ area/GP Ib^+^ area ratio (Figure 4A). Confocal imaging revealed that CEACAM-1 fluorescence was predominantly localized intracellularly in all experimental groups. CEACAM-1 staining appeared diffuse throughout the platelet interior, with no evident accumulation at the cell periphery (Figure 4B). Together, these findings indicate that in non-procoagulant platelets adhered to PLL, CEACAM-1 is predominantly localized intracellularly, while its relative abundance is increased by hyperglycemic conditions and reduced by ASA treatment.

### 3.5. Platelet Spreading on Fibrinogen

**Figure 5:**
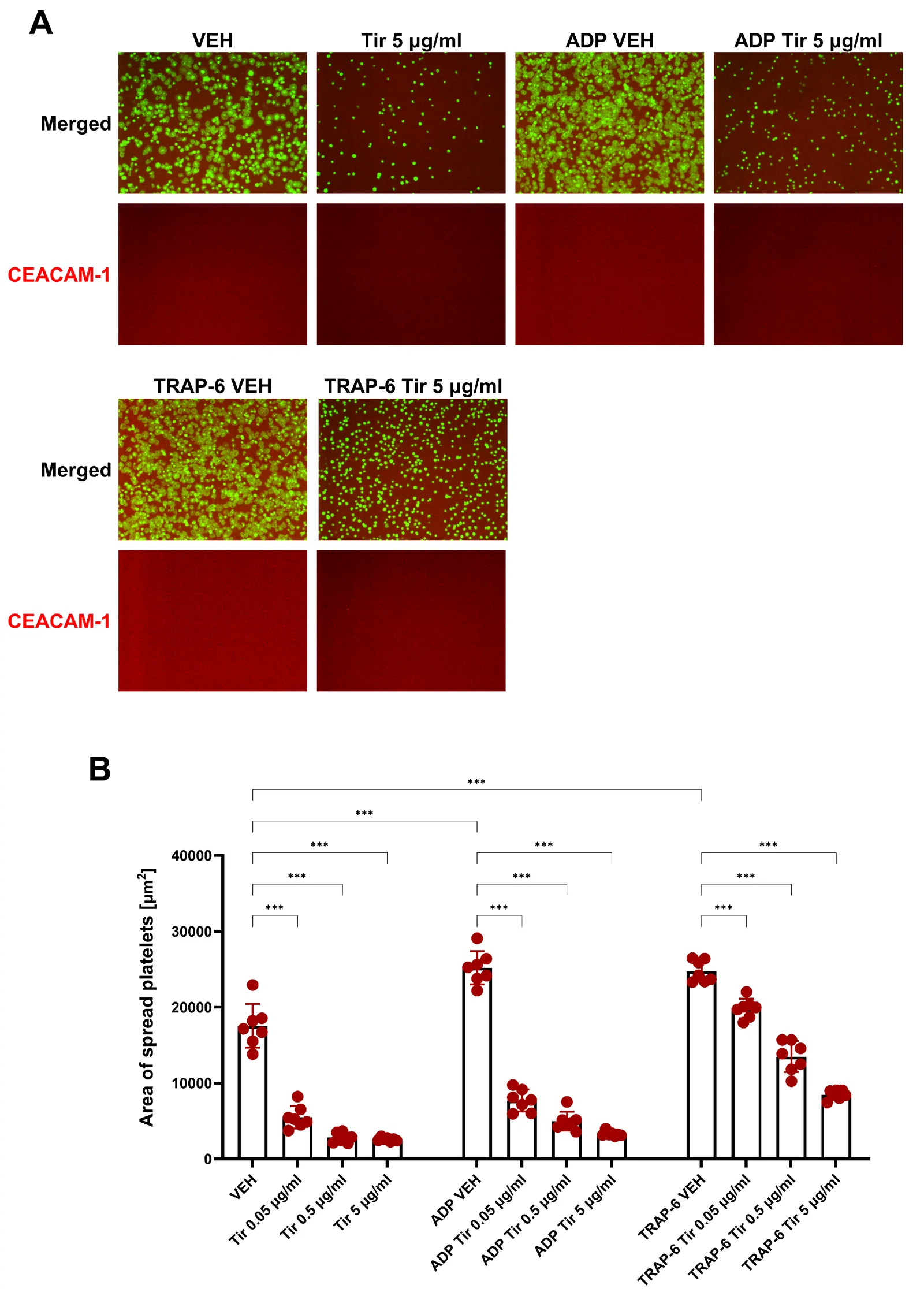
**Absence of detectable CEACAM-1 fluorescence in platelets spread on fibrinogen after tirofiban (Tir) treatment and ADP or TRAP-6 stimulation.** (**A**) Representative confocal images of platelets spread on fibrinogen-coated surfaces after tirofiban treatment and ADP or TRAP-6 stimulation. Columns represent the experimental groups, as indicated above the images. The top row shows merged images containing GP Ib fluorescence (green), used to identify platelets, and CEACAM-1 fluorescence (red). The bottom row shows CEACAM-1 fluorescence alone (red). No detectable CEACAM-1 fluorescence was observed in spread platelets across the experimental conditions. Magnification ×400. (**B**) Effect of tirofiban (Tir) on platelet spreading in unstimulated (VEH), ADP-treated, and TRAP-6-treated platelets. *** *p* < 0.001; *n* = 7 healthy volunteers. For each volunteer, the complete platelet-spreading experiment, including VEH, ADP, and TRAP-6 conditions with the corresponding tirofiban treatments, was performed. One field of view was analyzed for each condition, with each volunteer representing one biological replicate. Statistical significance was determined by Kruskal–Wallis test with Dunn’s multiple comparisons test.

On fibrinogen, platelets undergo spreading, characterized by cell flattening and the filopodia formation. Compared with adhesion to PLL, this model reflects a more pronounced morphological response to platelet activation, although PS exposure was not observed (Figure S4). Because platelet spreading on fibrinogen is αIIbβ3-dependent, tirofiban was included to inhibit this integrin-mediated process. CEACAM-1 fluorescence was undetectable in permeabilized spread platelets regardless of ADP or TRAP-6 stimulation and remained undetectable following αIIbβ3 inhibition with tirofiban (Figure 5A). Tirofiban reduced platelet spreading in untreated, ADP-stimulated, and TRAP-6-stimulated platelets. Together, these findings show that CEACAM-1 was not detectable by immunofluorescence in platelets spread on fibrinogen, irrespective of agonist stimulation or αIIbβ3 inhibition.

### 3.6. Western Blot Analysis

Western blot analysis confirmed the presence of CEACAM-1 in platelets spread on fibrinogen, irrespective of additional agonist stimulation with ADP or TRAP-6 or αIIbβ3 inhibition with tirofiban. CEACAM-1 was also detected in resting washed platelets used as reference controls. These findings confirm that the absence of detectable CEACAM-1 immunofluorescence in spread platelets does not reflect a lack of CEACAM-1 protein.

## 4. Discussion

In this study, CEACAM-1 exhibited a highly dynamic localization pattern that differed markedly between adherent, spread, aggregating, and procoagulant platelets. Importantly, the antibody used throughout the study recognizes the extracellular domain of CEACAM-1 according to the manufacturer’s specifications. Therefore, the detected fluorescence reflects not only receptor presence but also the availability of a functionally relevant extracellular region involved in homophilic interactions and signaling.

Platelet activation results in distinct functional states, including spread, aggregating, and procoagulant platelets. Procoagulant platelets are characterized by sustained cytosolic Ca^2+^ elevation, PS exposure, membrane ballooning, and reduced adhesive capacity. In contrast, aggregating platelets maintain membrane asymmetry and active αIIbβ3, enabling their participation in platelet aggregation and clot retraction [6]. Platelet spreading represents a distinct αIIbβ3-dependent adhesive response accompanied by extensive cytoskeletal rearrangement [12]. Confocal microscopy revealed CEACAM-1 fluorescence in PS-exposing regions of ADP-activated washed platelets displaying a procoagulant phenotype (Figure S3). Focal CEACAM-1 fluorescence was also observed at the platelet membrane, indicating localized accessibility of the antibody-recognized extracellular epitope. Furthermore, under low-calcium conditions, TRAP-6 induced the appearance of a P-selectin-positive population without detectable CEACAM-1-positive or ANX V-positive populations (Figure S6). This supports the possibility that increased CEACAM-1 surface availability in ADP-stimulated suspended platelets is associated with the procoagulant phenotype rather than with platelet activation per se. Previous studies have shown that CEACAM-1–actin interactions are regulated by Ca^2+^ and negatively charged phospholipids, including PS [13]. Although a direct mechanistic link cannot be established, this may provide a biological context for the observed spatial association between CEACAM-1 and PS exposure.

In contrast, within fibrin–platelet clots, where PS-exposing platelets are incorporated into the fibrin net, no detectable surface CEACAM-1 fluorescence was observed immediately after clot formation. CEACAM-1 became detectable only after overnight incubation and platelet permeabilization. Platelets exhibiting intracellular CEACAM-1 fluorescence frequently displayed an irregular morphology with pseudopodia-like extensions rather than the balloon-shaped appearance characteristic of procoagulant platelets, suggesting that the detected signal may originate from the aggregating platelet population. The delayed appearance of intracellular CEACAM-1 fluorescence may reflect processes occurring during the prolonged incubation period, including clot retraction, which involves extensive cytoskeletal rearrangement [14] and could alter the accessibility of the antibody-recognized CEACAM-1 domain. However, this interpretation remains speculative and requires direct experimental verification. 

**Figure 6:**
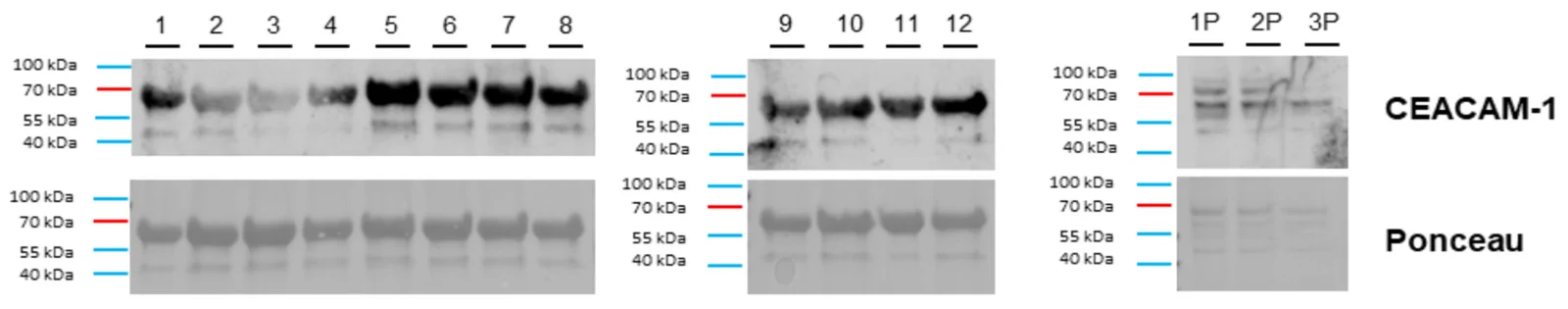
**Detection of CEACAM-1 in spread platelets.** Representative Western blot images showing CEACAM-1 in platelets spread on fibrinogen. Lanes 1–4 represent platelets without additional agonist stimulation, lanes 5–8 represent ADP-stimulated platelets, and lanes 9–12 represent TRAP-6-stimulated platelets. VEH-treated samples are shown in lanes 1, 5, and 9. Tirofiban was used at 0.05 µg/mL (lanes 2, 6, and 10), 0.5 µg/mL (lanes 3, 7, and 11), and 5 µg/mL (lanes 4, 8, and 12). Resting washed platelets were included as controls (lanes 1P–3P). The upper panels show CEACAM-1 immunoreactivity, whereas the lower panels show Ponceau S staining used to assess protein loading. The red and blue lines indicate the molecular-weight reference positions corresponding to the molecular-weight values shown next to the blots; the colors have no analytical significance. Representative blots from one healthy volunteer are shown. All experimental samples were analyzed as single samples, whereas resting washed platelets were analyzed in triplicate. The experiment was performed using platelets from three healthy volunteers (*n* = 3 biological replicates).

To evaluate CEACAM-1 localization specifically in non-procoagulant platelets, we used platelets spread on fibrinogen, as this condition is not associated with sustained intracellular Ca^2+^ concentration sufficient to induce PS exposure. Platelet spreading on fibrinogen is an αIIbβ3-dependent process accompanied by extensive cytoskeletal reorganization, granule secretion, and platelet flattening [15]. Following platelet permeabilization, no CEACAM-1 signal was detected in either the intracellular or extracellular compartments of spread platelets. This lack of signal was consistently observed after spreading alone as well as following stimulation with ADP, TRAP-6, or tirofiban treatment. To determine whether this reflected the loss of CEACAM-1 protein, Western blot analysis of spread platelets was performed (Figure 6). Detection of CEACAM-1 indicated that receptor shedding was unlikely to account for the absence of the microscopic signal. The mechanism underlying the loss of detectable CEACAM-1 signal during platelet spreading remains to be elucidated.

In platelets adhered to PLL, CEACAM-1 fluorescence was predominantly intracellular, and high-glucose conditions increased the CEACAM-1^+^ area/GP Ib^+^ area ratio. In parallel, ADP stimulation of platelets in suspension was associated with the appearance of a distinct CEACAM-1-positive population. Together, these observations raise the hypothesis that an intracellular pool of CEACAM-1 may contribute to increased surface availability during platelet activation. However, direct redistribution of CEACAM-1 from intracellular compartments to the platelet surface was not assessed and remains to be established. 

Thrombi formed on collagen fibres under flow contain platelets in different activation states, including aggregating and procoagulant platelets [7]. In our study, CEACAM-1 showed distinct localization patterns depending on platelet activation state. Confocal imaging of isolated procoagulant platelets revealed a marked spatial association between CEACAM-1 and PS-exposing regions, whereas this relationship was less apparent within thrombi formed under flow. Given the functional platelet heterogeneity in thrombi, the overall CEACAM-1 signal may represent the combined contribution of platelet populations with different patterns of CEACAM-1 localization.

An important limitation of the present study is that the CEACAM-1L and CEACAM-1S isoforms were not distinguished. The antibodies used were generated against recombinant CEACAM-1 constructs encompassing the extracellular region (human CEACAM-1 Gln35–Gly428, accession P13688; mouse CEACAM-1 Met1–Gly428, accession P31809) and therefore do not provide information on the alternatively spliced cytoplasmic tail. Consequently, the CEACAM-1 signal detected in our experiments cannot be assigned specifically to CEACAM-1L, CEACAM-1S, or both isoforms. Isoform-specific analysis will be required to resolve this issue in future studies.

The present study is the first to demonstrate distinct patterns of platelet CEACAM-1 localization, particularly differences in the availability of its extracellular domain across different platelet activation states. Further studies are required to determine whether these differences have functional consequences for CEACAM-1 activity and whether its role varies with platelet functional state or the local microenvironment defined by platelet–platelet, or platelet–fibrin interactions. These findings provide a basis for investigating whether CEACAM-1 localization represents a previously unexplored, context-dependent mechanism affecting platelet activity.

## Data Availability

The data that support the findings of this study are available on request from the corresponding author. The data are not publicly available due to privacy or ethical restrictions.

## Supplementary Materials

The following supporting information can be downloaded at https://www.mdpi.com/article/10.3390/cells15211920/s1, Figure S1: Representative flow cytometry dot plots of unstimulated control platelets under normoglycemic and hyperglycemic (GLU) conditions and after ASA treatment. Experimental groups are indicated to the left of the dot plots. The horizontal axis represents GP Ib fluorescence detected in the FITC channel. In the left panel, the vertical axis represents CEACAM-1 fluorescence detected in the APC channel, whereas in the right panel it represents ANX V fluorescence detected in the APC channel. In unstimulated samples, two platelet populations differing in GP Ib fluorescence were distinguishable, whereas no distinct population characterized by increased APC fluorescence was observed; Figure S2: Representative flow cytometry dot plots of ADP-stimulated platelets from the VEH group. Platelets were stained with GP Ib without the addition of the Alexa Fluor 647-conjugated anti-CEACAM-1 antibody (A) or Alexa Fluor 647-conjugated ANX V (B). The horizontal axis represents GP Ib fluorescence detected in the FITC channel, and the vertical axis represents fluorescence detected in the APC channel. In both controls, no distinct population characterized by high APC fluorescence was observed; Figure S3: Representative confocal images of ADP-activated platelets. Platelets were stained with ANX V (green) and CEACAM-1 (red). Merged images demonstrate the spatial association of ANX V and CEACAM-1 signals. (A) Upper row: a representative balloon-shaped platelet. Lower row: balloon-shaped platelets exposing PS. (B) Focal accumulation of CEACAM-1 fluorescence; Figure S4: Representative confocal images of fibrin-platelet clots immediately after clot formation under normoglycemic and hyperglycemic (GLU) conditions and following treatment with tirofiban (Tir). Individual experimental groups are shown in separate columns, whereas rows display merged, CEACAM-1 fluorescence channel, and fibrin net. No detectable intracellular or extracellular CEACAM-1 signal was observed in any experimental group immediately after clot formation; Figure S5: Representative confocal images of spread platelets stained with ANX V and GP Ib. The upper row shows merged images, the middle row shows the ANX V fluorescence channel (red), and the lower row shows the GP Ib fluorescence channel identifying spread platelets. No detectable ANX V signal was observed in spread platelets; Figure S6: Representative flow cytometry dot plots of platelets activated with TRAP-6 in the presence of low calcium concentration (1 mM CaCl_2_). Platelets were identified using GP Ib staining. The horizontal axis indicates GP Ib fluorescence in the FITC channel, whereas the vertical axis represents APC fluorescence corresponding to CEACAM-1, ANX V, or P-selectin, as indicated on each dot plot.

## Abbreviations

ADP	adenosine diphosphate
ANX V	annexin V
ASA	acetylsalicylic acid
BSA	bovine serum albumin
CaCl_2_	calcium chloride
CEACAM-1	carcinoembryonic antigen-related cell adhesion molecule 1
FcRγ	Fc receptor gamma chain
GLU	hyperglycemic condition
GP Ib	glycoprotein Ib
HEPES	4-(2-hydroxyethyl)-1-piperazineethanesulfonic acid
ITAM	immunoreceptor tyrosine-based activation motif
ITIM	immunoreceptor tyrosine-based inhibitory motif
ITSM	immunoreceptor tyrosine-based switch motif
MgCl_2_	magnesium chloride
PBS	phosphate-buffered saline
PFA	paraformaldehyde
PECAM-1	platelet endothelial cell adhesion molecule 1
PLL	poly-L-lysine
PPACK	D-phenylalanyl-L-prolyl-L-arginine chloromethyl ketone
PPP	platelet poor plasma
PRP	platelet-rich plasma
PS	phosphatidylserine
SDS	sodium dodecyl sulfate
SEM	standard error of the mean
Tir	tirofiban
TRAP-6	thrombin receptor-activating peptide-6
VEH	vehicle
