# Supplementary material for: Differential Localization of CEACAM-1 in Distinct Platelet Populations
Source: Cells. 2026 Oct 22;15(21):1920. doi: 10.3390/cells15211920 (PMC13761983; doi:10.3390/cells15211920)

Supplementary Materials:

Figure S1. Representative flow cytometry dot plots of unstimulated control platelets under normoglycemic and hyperglycemic (GLU) conditions and after ASA treatment. Experimental groups are indicated to the left of the dot plots. The horizontal axis represents GP Ib fluorescence detected in the FITC channel. In the left panel, the vertical axis represents CEACAM-1 fluorescence detected in the APC channel, whereas in the right panel it represents ANX V fluorescence detected in the APC channel. In unstimulated samples, two platelet populations differing in GP Ib fluorescence were distinguishable, whereas no distinct population characterized by increased APC fluorescence was observed.

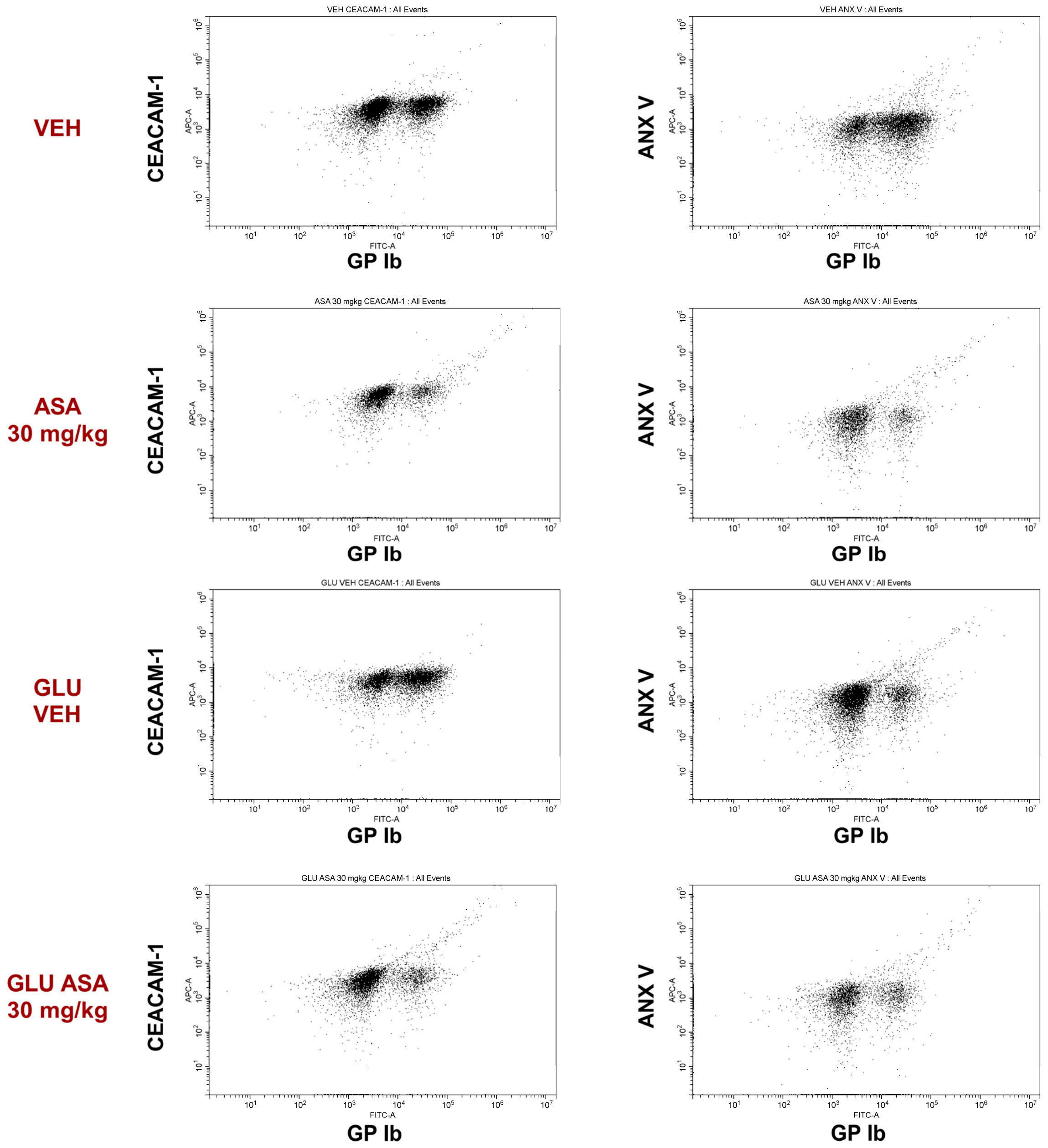

Figure S2. Representative flow cytometry dot plots of ADP-stimulated platelets from the VEH group. Platelets were stained with GP Ib without the addition of the Alexa Fluor 647-conjugated anti-CEACAM-1 antibody (A) or Alexa Fluor 647-conjugated ANX V (B). The horizontal axis represents GP Ib fluorescence detected in the FITC channel, and the vertical axis represents fluorescence detected in the APC channel. In both controls, no distinct population characterized by high APC fluorescence was observed.

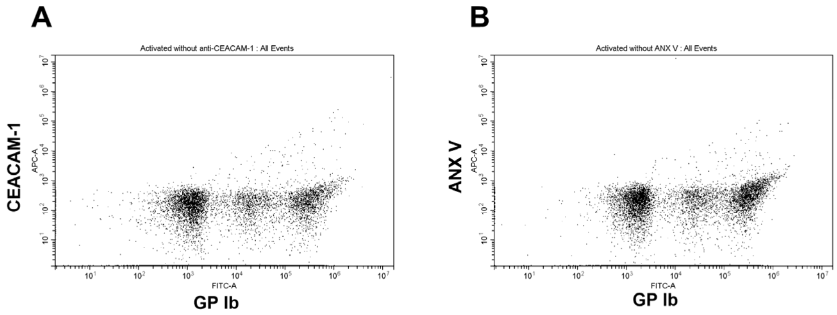

Figure S3. Representative confocal images of ADP-activated platelets. Platelets were stained with ANX V (green) and CEACAM-1 (red). Merged images demonstrate the spatial association of ANX V and CEACAM-1 signals. (A) Upper row: a representative balloon-shaped platelet. Lower row: balloon-shaped platelets exposing PS. (B) Focal accumulation of CEACAM-1 fluorescence.

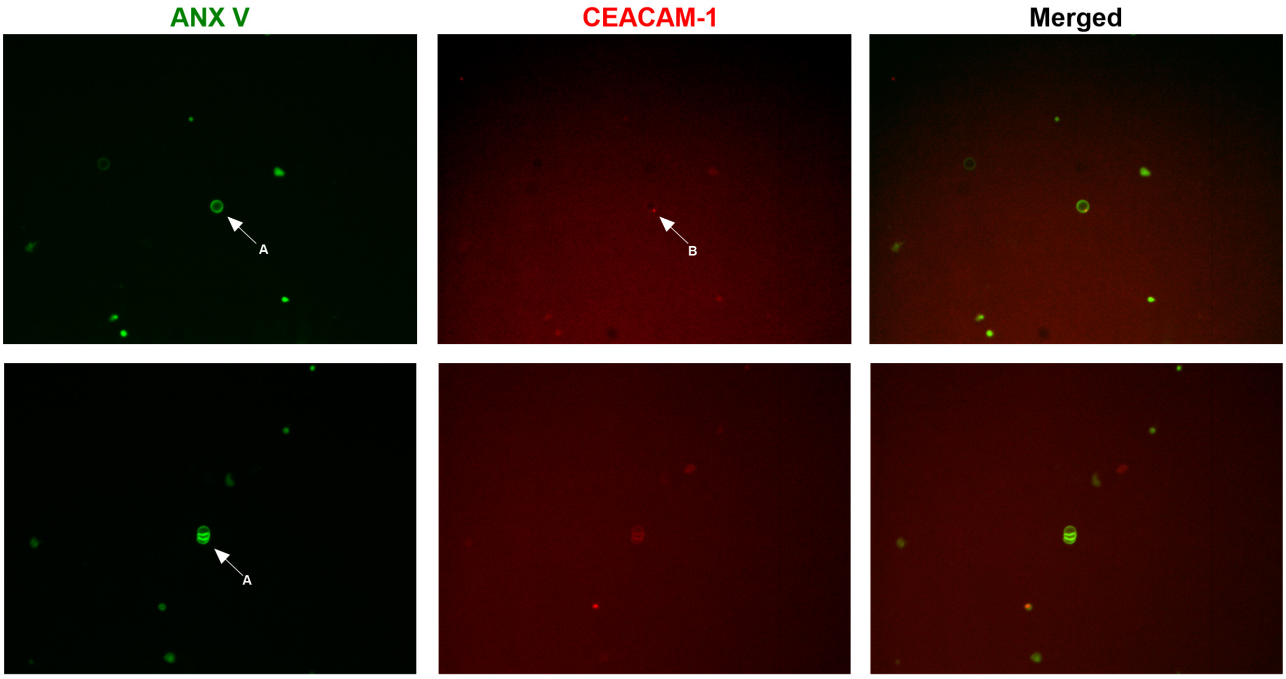

Figure S4. Representative confocal images of fibrin-platelet clots immediately after clot formation under normoglycemic and hyperglycemic (GLU) conditions and following treatment with tirofiban (Tir). Individual experimental groups are shown in separate columns, whereas rows display merged, CEACAM-1 fluorescence channel, and fibrin net. No detectable intracellular or extracellular CEACAM-1 signal was observed in any experimental group immediately after clot formation.

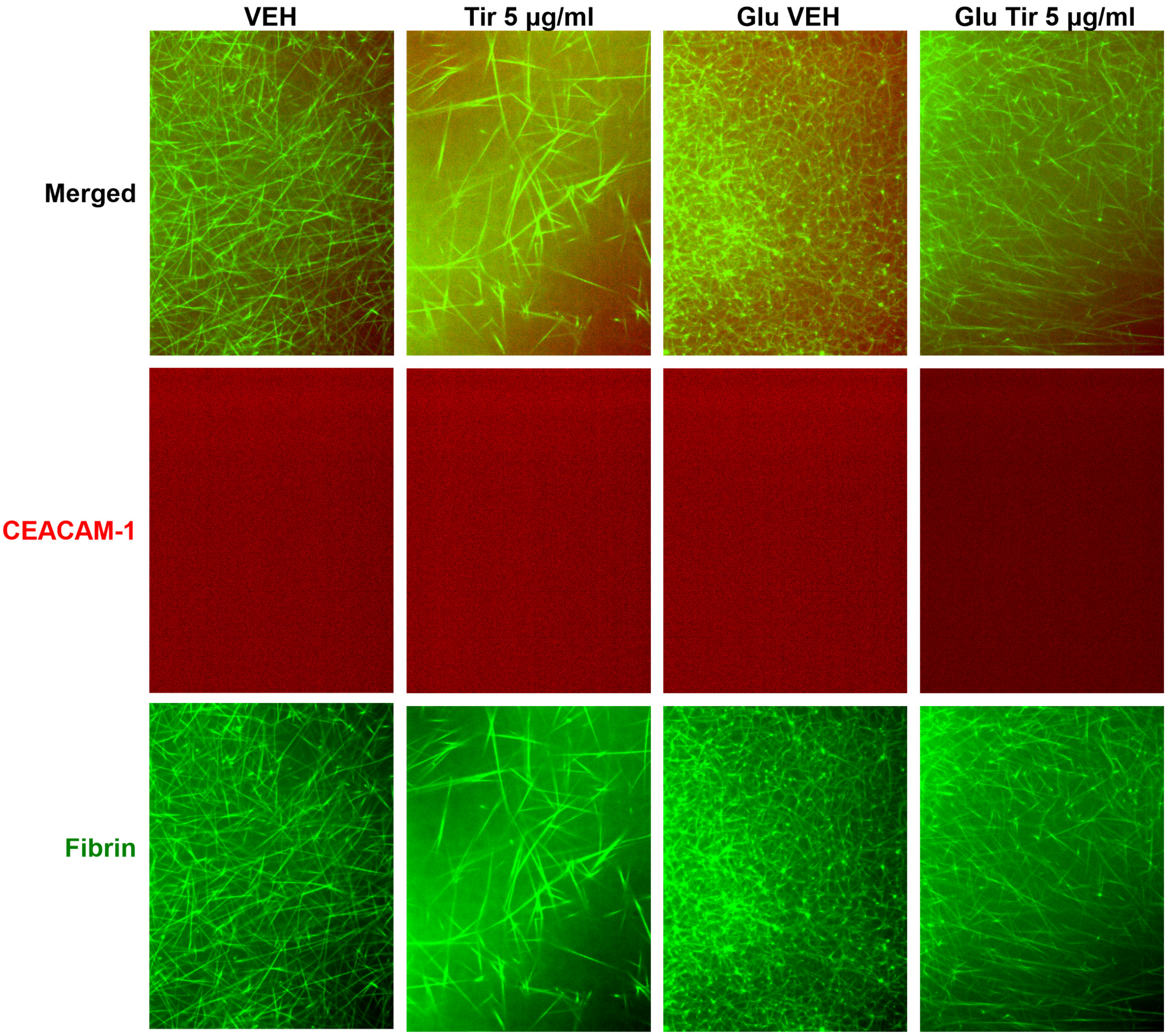

Figure S5. Representative confocal images of spread platelets stained with ANX V and GP Ib. The upper row shows merged images, the middle row shows the ANX V fluorescence channel (red), and the lower row shows the GP Ib fluorescence channel identifying spread platelets. No detectable ANX V signal was observed in spread platelets.

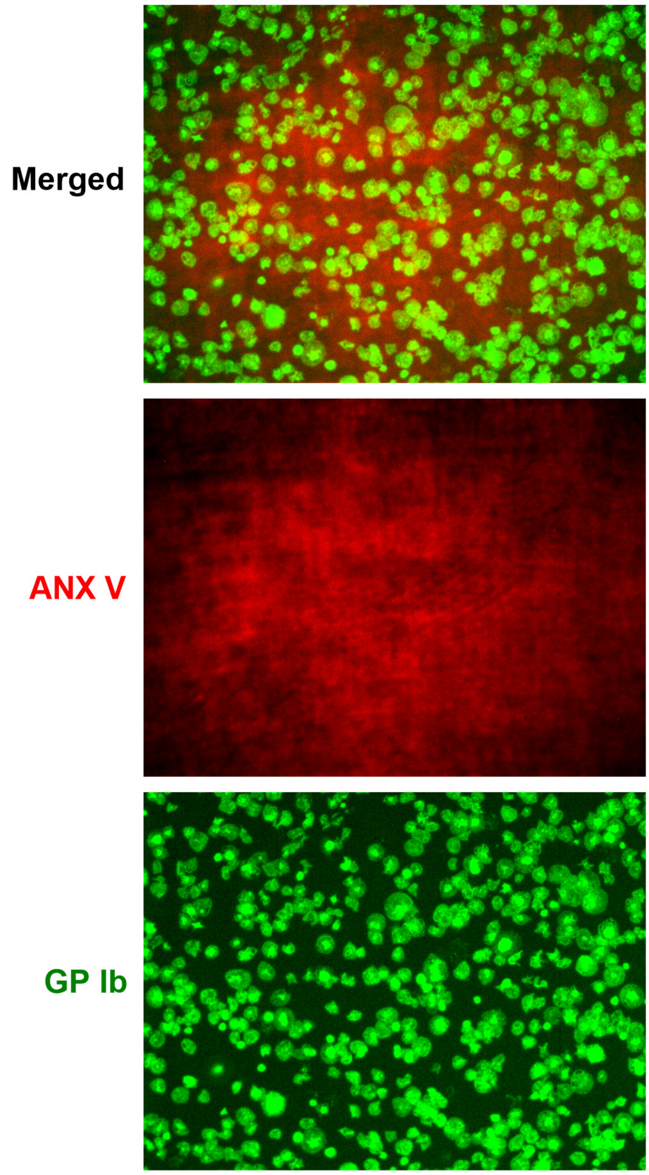

Figure S6. Representative flow cytometry dot plots of platelets activated with TRAP-6 in the presence of low calcium concentration (1 mM $CaCl_2$). Platelets were identified using GP Ib staining. The horizontal axis indicates GP Ib fluorescence in the FITC channel, whereas the vertical axis represents APC fluorescence corresponding to CEACAM-1, ANX V, or P-selectin, as indicated on each dot plot.

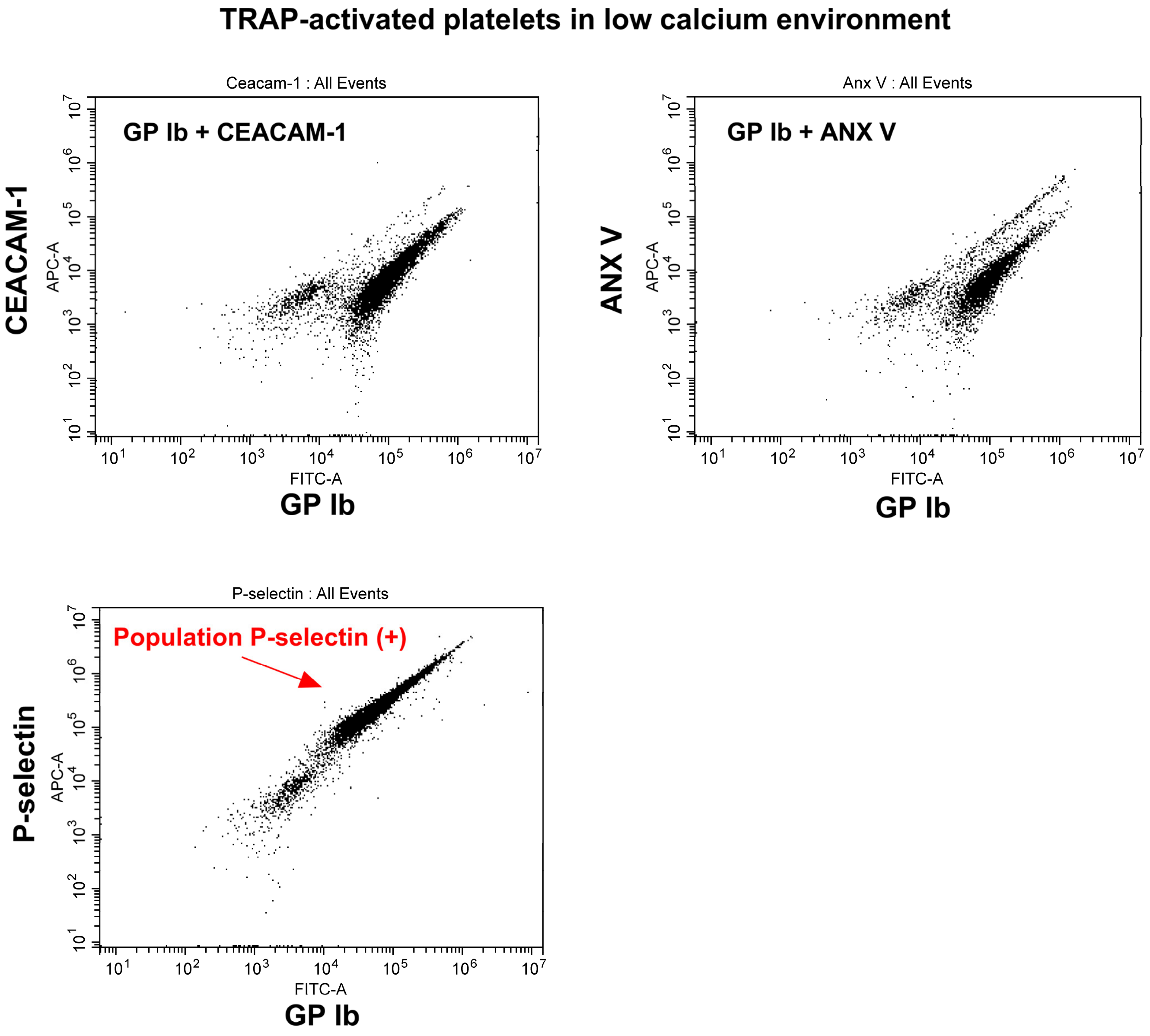

Supplement: Supplementary file 1 [file cells-15-01920-s001.zip › cells-4545813-supplementary.pdf]
